# Antiretroviral therapy programme outcomes at Senkatana antiretroviral therapy clinic, Lesotho: a four-year retrospective cohort study

**DOI:** 10.11604/pamj.2023.46.74.40122

**Published:** 2023-11-02

**Authors:** Mwamba Kabuya, Alfred Musekiwa, Simbarashe Takuva, Lehana Thabane, Lawrence Mbuagbaw

**Affiliations:** 1 Senkatana Antiretroviral Therapy (ART) Clinic Maseru, Maseru, Lesotho,; 2 School of Health Systems and Public Health, Faculty of Health Sciences, University of Pretoria, Pretoria, South Africa,; 3 Department of Health Research Methods, Evidence and Impact, McMaster University, Hamilton, Ontario, Canada,; 4 Biostatistics Unit, Father Sean O´Sullivan Research Centre, St Joseph´s Healthcare Hamilton, Hamilton, Ontario, Canada

**Keywords:** Adherence, viral load suppression, ART treatment outcomes

## Abstract

**Introduction:**

sub-Saharan Africa, home to over 10% of the world´s population, is the worst Human Immunodeficiency Virus (HIV)-affected region in the world. HIV/AIDS is a major public health challenge in Lesotho, with an HIV prevalence of 25.6% in 2018. The aim of this study was to evaluate the treatment outcomes of people living with HIV (PLHIV) on antiretroviral therapy (ART) after 48 months of initiation.

**Methods:**

we conducted a register-based retrospective cohort study for all patients registered at the Senkatana ART Clinic from January to December 2014 and followed them for 48 months until 2018. The ART treatment register and treatment cards were the primary source of data. Data were captured and cleaned in Epi info version 7 and exported into Stata version 14 for analysis. Descriptive statistics were used to describe participant characteristics. Due to the lack of incident data, the factors associated with treatment outcomes were determined using Chi-square tests and logistic regression.

**Results:**

in 2014, 604 patients were enrolled on ART, of which the majority were female (59.4%) and married (54.8%). The mean age (standard deviation (SD)) at which ART was started was 36 years (10.5) years. After 48 months of initiation, the cohort consisted of 387 patients of which 365 (94.3%) were retained on treatment. In the multivariable logistic regression model, neither demographic characteristics nor clinical factors were associated with ART treatment outcome (viral load suppression, adherence, or ART retention), however, the univariable analysis showed that higher CD4 count at initiation was associated with viral load suppression.

**Conclusion:**

retention, viral load suppression, and adherence were generally good in this cohort after 48 months of initiation. CD4 at initiation was a significant predictor of viral load suppression at 48 months. The ART programme has managed to maintain high viral load suppression and improve immunity in patients who are immunocompromised. Proper data quality management is required for adequate patient monitoring to enable clinical personnel to record and use individual patient data for guiding the clinical management of such patients. Strengthening patient support and tracing will help to reduce the number of patients lost to follow-up.

## Introduction

Sub-Saharan Africa, home to just about 10% of the world´s population, remains the worst HIV-affected region in the world. In fact, this geographical area is home to approximately 70% of all people living with HIV (PLHIV) globally [1]. According to the 2018 Lesotho Population-Based HIV Impact Assessment (LePHIA), the prevalence of HIV was estimated to be 25.6%, which is the second highest country prevalence in the world. The annual incidence was 1.1% translating to 10,000 new HIV infections among adults 15 - 59 years annually. Since HIV is the leading cause of premature death, it has contributed to Lesotho's reporting of the second shortest life expectancy of 45 years at birth among 195 countries and territories [2].

The introduction of antiretroviral therapy (ART) for the treatment of HIV in 2004 has led to massive reductions in mortality and slowed the progression of the disease and transmission of infection [3]. According to LePHIA 2020, Lesotho has now met all the joint United Nations Programme on HIV/AIDS (UNAIDS) 90-90-90 targets among adults (ages 15 years and older) living with HIV. The country has surpassed the overall target for 2020 to have more than 73% of all adults living with HIV achieving viral load suppression (VLS). Although the country´s HIV programme has made great strides, gaps remain. For instance, HIV prevalence was higher among women than men. It was more than five times higher among young women aged 20-24 years than their male counterparts. In addition, VLS among young people remained below the UNAIDS targets, regardless of sex [4]. Even though the country had done well in their UNAIDS target generally, but when doing data extrapolation in terms of age and sex, we observe that young children have not achieved the third UNAIDS target which is VLS, hence the problem for the country. The same report shows that the country´s new infections are declining but 80% of those new infections are from the same younger age group. In order to strengthen the ART programme, an evaluation of ART treatment outcomes in Lesotho is highly needed.

**Study aim and objective:** the aim of this study was to evaluate the treatment outcomes of HIV patients on ART after 48 months of initiation. Specific objectives: A) To describe the profile of patients who were initiated on first-line ART at Senkatana ART Clinic, Lesotho, between 1^st^ January and 31^st^ December, 2014; B) to determine factors associated with viral load suppression after 48 months post ART initiation until 2018; C) to determine the factors associated with adherence at 48 months post-ART initiation until 2018; D) to determine the factors associated with ART outcomes (retention in care and attrition ) after 48 months post ART attrition until 2018.

## Methods

**Study design:** this was a register-based retrospective cohort study for all patients enrolled at the Senkatana ART clinic from January to December 2014.

**Study setting:** this retrospective cohort study was conducted at Senkatana ART Clinic, in Maseru city, capital of Lesotho in Lithabaneng village. Senkatana ART Clinic is the main national referral facility for PLHIV and tuberculosis (TB) in Maseru. It offers the following services: HIV/AIDS, TB services, and cervical cancer screening. It is serving an estimated population of 28,371 in Maseru District.

**Study population:** all PLHIV registered and initiated on ART at Senkatana between 1^st^ January 2014 and 31^st^ December 2014 form our study population. After inclusion criteria, 604 patients were included in the study and followed for 48 months until 2018.

**Inclusion criteria:** all adult ART-naïve PLHIV (age ≥15 years) registered and initiated on first-line ART at Senkatana between 1^st^ January 2014 and 31^st^ December 2014 were included in the study. Transfer in from other facilities with transfer documents who fall within this cohort were also included.

**Exclusion criteria:** all patient records that did not provide the required information in the register or ART card were excluded in the analysis. A total of 48 files were excluded in the study and this includes missing files, incomplete information, outcome not stated, and transfer out.

**Sampling method:** since this was a record review study to assess ART treatment outcomes of all patients who were registered and initiated on ART from 1^st^ January 2014 to 1^st^ December 2014, no sampling method was required as all registered ART patients were included in the analysis.

**Data collection:** data on patient´s information such as demographic and clinical characteristics were extracted from the ART register and ART card, and then entered into a developed database using Epi info version 7.

**Data analysis:** extracted data were exported into a Microsoft Excel spreadsheet, cleaned, and thereafter imported into Stata version 17.0 statistical software for analysis. Descriptive frequency tables were created for categorical variables and continuous data were presented as means with standard deviations (SD´s) and the results were reported as per objective. Bivariate analysis was used to determine the strength of associations between demographic characteristics and viral load, adherence, and treatment outcomes. Due to a lack of incident data, we used the Pearson´s Chi-square test and multivariable logistic regression model to identify factors independently associated with ART outcomes and presented results as crude odds ratios (OR) with 95% confidence intervals (CI) and p-values. A significance level of 0.05 was used.

**Ethical consideration:** ethics approval was obtained from the National Health Research Committee of the Lesotho Ministry of Health (ID04-2020) before data collection. Permission was sought from Senkatana ART Clinic to gain access to patients´ information. Confidentiality was maintained throughout the study. Anonymity was maintained from data collection to the end of the study.

**Operational definitions**

**ART regimens:** 1) TDF-3TC-EFV: Tenofovir-Lamivudine-Efavirenz; 2) AZT-3TC-NVP: Zidovudine-Lamivudine-Niverapine; 3) AZT-3TC-EFV: Zidovudine-Lamivudine-Efavirenz; 4) ABC-3TC-EFV: Abacavir-Lamivudine- Efavirenz. Adherence: the ART adherence was assessed by pill counts at different time intervals for study period. Reported with good adherence if as taking ≥ 95% of their pills and poor adherence if as taking <95% and >105% of their pills at different time intervals. Viral load suppression (VLS): reduction of HIV viral load to an undetectable level. In Lesotho, optimal ART regimens should maintain clients with a viral load < 50 cps/ml.

## Results

**Cohort characteristics:** a total of 604 patients initiated ART between January to December 2014 at Senkatana ART Clinic. Of the 604 patients, the majority were female (359, 59.4%) and more than half were married (54.8%). More patients were in the 30-34 years age group (140, 23,2%) (Table 1). Regarding the functional status of the patients, (79.6%) were working at baseline. Almost 40% of the patients were in World Health Organization (WHO) stage III (38.3%). The median baseline CD4 cell count was at 248 (IQR: 115 - 369) for patients with documented CD4 results. Regarding TB prophylaxis and prevention of opportunistic infection, 77.5% were on isoniazid (INH) for prevention of TB and 91.2 % did take co - trimoxazole (CTX). A total of 109 (18.0%) ART patients were co-infected with TB. Furthermore, a total of 583 patients were screened for sexually transmitted infections (STIs), of which 27 (4.6%) showed signs of STIs (Table 2).

**Table 1:** demographic characteristics of cohort at baseline (n=604)

Variable	Frequency (n)	Percentage
**Sex**		
Female	359	59.4%
Male	245	40.6%
**Marital status**		
Married	330	54.8%
Separated	55	9.1%
Divorced	11	1.8%
Single	134	22.3%
Widowed	72	12.0%
**Age at initiation**		
17-19	10	1.7%
20-24	52	8.6%
25-29	97	16.1%
30-34	140	23.2%
35-39	113	18.7%
40-44	80	13.2%
45-49	45	7.5%
50-54	30	5.0%
55-59	13	2.2%
60+	24	4.0%

Note: marital status-missing value of 2

**Table 2:** clinical characteristics of cohort at baseline (n=604)

Variable	Frequency (n)	Percentage
**Weight at initiation (Kg)**		
<45	79	13.1%
46-55	199	33.1%
56-65	191	31.7%
>65	133	22.1%
**Functional status**		
Work	480	79.6%
Ambulatory	121	20.1%
Bedridden	2	0.3%
**WHO clinical stage**		
Stage I	186	31.3%
Stage II	165	27.7%
Stage III	228	38.3%
Stage IV	16	2.7%
**CD4 count at initiation**		
Low, <100	121	22.0%
Medium, 101-350	270	49.0%
High, >350	160	29.0%
**On INH**		
Missing	22	3.6%
No	114	18.9%
Yes	468	77.5%
**CTX**		
Missing	20	3.3%
No	33	5.5%
Yes	551	91.2%
**Co-infection (TB status at initiation)**		
Missing	20	3.3%
No	475	78.6%
Yes	109	18.0%
**STI screening**		
No signs	556	95.4%
Signs	27	4.6%

Weight at initiation (Kg)-missing value of 2; functional status-missing value of 1; WHO clinical stage-missing value of 9; CD4 count at initiation-missing value of 53; STI screening-missing value of 21; CTX: co-trimoxazole; LFTU: loss to follow-up; WHO: World Health Organization; STI: sexually transmitted infection; INH: isoniazid; TB: tuberculosis

**Figure 1:**
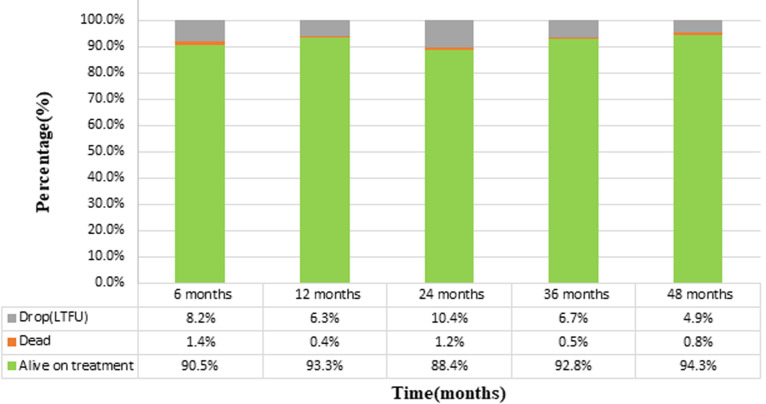
2014 anti-retroviral therapy (ART) cohort outcomes at Senkatana

**Anti-retroviral therapy (ART) cohort treatment outcomes:** it should be noted that data was for the same patients at different time intervals from 6 Months (2014), 12 months (2015), 24 months (2016), 36 months (2017) and 48 months (2018). We analysed data using a combination of longitudinal and cross-sectional methods, therefore the overall retention remained at 94.3%. The proportion of patients alive on treatment (retention) was at 94.3% in 2018 (48 months after initiation) with a mortality rate (deaths) of 0.8% and loss to follow-up (LTFU) at 4.9% at Senkatana. Retention stayed above 80% since 2014. Mortality outcome is based on the report found in the register, which is the same as lost to follow-up. The country uses a verbal autopsy system from the community through the village health workers who are following up with clients regularly for drug pick or for other reasons. There is also a network of ART community through community ART group formed in all villages who were also providing the information. Once the messages were received, the register or ART card would indicate “dead” in the outcome section (Figure 1).

**Factors associated with ART retention:** in univariate analyses, both the Chi-square test and the logistic regression analyses showed no association between sex, age, marital status, WHO clinical stage, functional status, co-infection with TB, and CD4 count, with ART retention at 48 months after initiation (Table 3).

**Table 3:** demographic factors and clinical factors related to ART treatment outcomes and Chi-square test (n=387)

Variables	ART Treatment Outcomes		Total	Adjusted OR (95% CI)	P-Value	X^2^	P-Value
	Attrition	Retention					
**Sex**							
Female	13(5.6)	219(94.4)	232	1.04(0.43-2.49)	0.933	0.01	0.933
Male	9(5.8)	146(94.2)	155	Ref	-		
**Age group**							
< 35	12(6.6)	171(93.4)	183	Ref		2.74	0.253
35-50	6(3.7)	157(96.3)	163	1.83(0.67-5.01)	0.235		
> 50	4(9.8)	37(90.2)	41	0.64(0.19-2.12)	0.475		
**Marital status**							
Married	13(5.7)	216(94.3)	229	1.01(0.42-2.42)	0.982	0.001	0.982
Unmarried	9(5.7)	148(94.3)	157	Ref	-		
**WHO clinical stage**							
Stage I & II	14(6.0)	219(94.0)	233	Ref		0.07	0.782
Stage III & IV	8(5.3)	142(94.7)	150	1.13(0.46-2.77)	0.782		
**Functional status**							
Work	19(6.1)	293(93.9)	312	0.65(0.18- 2.26)	0.500	0.46	0.497
Ambulatory	3(4.1)	71(95.9)	74	Ref	-		
**Co-infection (TB status at initiation)**							
No	20(6.6)	284(93.4)	304	Ref		1.37	0.241
Yes	2(2.9)	67(97.1)	69	2.35(0.53-10.34)	0.255		
**CD4 count at initiation**							
Low, <100	3(4.1)	71(95.9)	74	Ref		0.71	0.701
Medium,101-350	8(4.6)	165(95.4)	173	0.87(0.22-3.31)	0.842		
High, >350	7(6.5)	100(93.5)	107	0.60(0.150-2.41)	0.475		

ART: anti-retroviral therapy; WHO: World Health Organization; TB: tuberculosis

**Figure 2:**
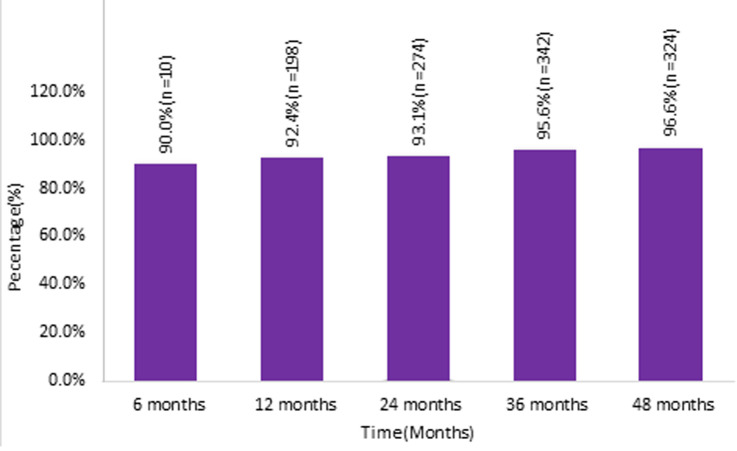
viral load suppression among 2014 cohort at Senkatana

**Viral load outcomes:** at 48 months, 324 (96.6%) patients achieved viral load suppression (Figure 2). In multivariable analyses, there was no association between sex, age, marital status, weight, selected clinical variables such as WHO clinical stage, functional status, and co-infection with TB with viral load suppression. However, the univariate analysis test showed that higher CD4 count at initiation was associated with viral load suppression (Table 4).

**Table 4:** demographic and clinical factors related to viral load suppression among ART patient Chi-square test (n=324)

Variables	Viral load suppression		Total	OR (95% CI)	P-value	X^2^	P-value
	Viral suppressed	Not virally suppressed					
**Sex**							
Female	187(97.4)	5(2.6)	192	1.78(0.53-5.96)	0.349	0.89	0.343
Male	126(95.5)	6(4.5)	132	Ref	-		
**Age group**							
< 35	144(94.7)	8(5.3)	152	Ref		3.27	0.194
35-50	138(98.6)	2(1.4)	140	3.83 (0.79 -18.36)	0.093		
> 50	31(96.9)	1(3.1)	32	1.72(0.20-14.27)	0.614		
**Marital status**							
Married	180(95.7)	8(4.3)	188	0.50(0.13 -1.94)	0.323	1.01	0.315
Unmarried	133(97.8)	3(2.2)	136	Ref	-		
**Weight at initiation (Kg)**							
<45	30(96.8)	1(3.2)	31	Ref		3.09	0.378
46-55	104(95.4)	5(4.6)	109	0.69(0.77-6.16)	0.743		
56-65	104(99.1)	1(0.9)	105	3.46(0.21-57.08)	0.384		
>65	74(94.9)	4(5.1)	78	0.61(0.06 -5.74)	0.671		
**WHO clinical stage**							
Stage I & II	186(96.9)	6(3.1)	192	Ref	-	0.14	0.707
Stage III & IV	123(96.1)	5(3.9)	128	0.79(0.23-2.67)	0.708		
**Functional status**							
Work	249(96.1)	10(3.9)	259	0.39(0.04-3.14)	0.380	0.82	0.364
Ambulatory	63(98.4)	1(1.6)	64	Ref	-		
**Co-infection (TB status at initiation)**							
No	246(97.2)	7(2.8)	253	Ref		2.17	0.140
Yes	56(93.3)	4(6.7)	60	0.39(0.11-1.40)	0.153		
**CD4 count at initiation**							
Low, <100	61(98.4)	1(1.6)	62			6.91	0.032*
Medium,101-350	140(93.9)	9(6.1)	149	0.25(0.03-2.05)	0.200		
High, >350	87(100)	0	87		-		

WHO: World Health Organization; TB: tuberculosis

**Factors associated with adherence:** univariate analyses showed that there was no association between sex, age, marital status, weight, WHO clinical stage, viral load assessment, functional status, co-infection with TB, and CD4 count, with adherence at 48 months after initiation (Table 5).

**Table 5:** demographic and clinical factors related to adherence among ART patients (n=211)

Variables	Adherence		Total	OR (95% CI)	P-value	X^2^	P-value
	Good	Poor					
**Sex**							
Female	125(93.9)	8(6.0)	133	1.54 (0.53-4.42)	0.422	0.65	0.419
Male	71(91.0)	7(8.9)	78	Ref	-		
**Age group**							
< 35	91(92.9)	7(7.1)	98	Ref		0.41	0.814
35-50	88(93.6)	6(6.4)	94	1.12 (0.36 -3.48)	0.834		
> 50	17(89.5)	2(10.5)	19	0.65(0.12-3.42)	0.615		
**Marital status**							
Married	117(95.1)	6(4.9)	123	2.25 (0.77-6.57)	0.138	2.29	0.130
Unmarried	78(89.7)	9(10.3)	87	Ref	-		
**Weight at initiation (Kg)**							
<45	18(94.7)	1(5.3)	19	Ref		0.84	0.838
46-55	74(91.4)	7(8.6)	81	0.58(0.06-5.08)	0.629		
56-65	57(95.0)	3(5.0)	60	1.06(0.10 -10.78)	0.964		
>65	46(92.0)	4(8.0)	50	0.63(0.07 - 6.11)	0.697		
**WHO clinical stage**							
Stage I& II	117(91.4)	11(8.6)	128	Ref		0.99	0.319
Stage III& IV	77(95.0)	4(4.9)	81	1.80 (0.56-5.88)	0.324		
**Viral load assessment**							
Not viral suppressed	7(77.9)	2(22.2)	9	Ref		3.26	0.071
Viral suppressed	176(93.6)	12(6.4)	188	4.19(0.78-22.41)	0.094		
**Functional status**							
Work	161 (91.4)	15 (8.5)	176			3.21	0.073
Ambulatory	35 (100)	0	35	-	-		
**Co-infection (TB status at initiation)**							
No	152(92.7)	12(7.3)	164	Ref		0.23	0.628
Yes	37(94.9)	2(5.1)	39	1.46(0.31-6.80)	0.630		
**CD4 count at initiation**							
Low, <100	37(90.2)	4(9.8)	41	Ref		2.19	0.333
Medium, 101-350	81(92.1)	7(7.9)	88	1.25(0.34-4.53)	0.733		
High, >350	63(96.9)	2(3.1)	65	3.40(0.59-19.50)	0.169		

WHO: World Health Organization; TB: tuberculosis

## Discussion

This study evaluated the treatment outcomes of people living with HIV on ART after 48 months of initiation. Our findings indicate that retention in care, viral load suppression, and adherence were generally good among this cohort after 48 months of initiation.

The rate of retention in care at 48 months after initiation was at 94.3%, which was higher than the rate of 83.9% reported in Tanzania [5], 55% in South Africa [6], and 91.4% in Mozambique [7]. Several possible reasons for high rates of retention in care in our study include: patients centered treatment environment, health education, and HIV status disclosure strategies. Further, there is a strong network of PLHIV that supports peer adherence to medication including home visits, awareness and education activities in the communities.

Our study showed that socio-demographic characteristics were not associated with retention in care. We report no differences in retention among female and male participants. Similar findings were observed in a mixed methods analysis conducted in Uganda and Kenya [8], whereas studies in Cameroon and India reported that male patients had poorer retention in care as compared to their female counterparts [9,10]. Research from Kenya and Ethiopia have reported divergent ART outcomes on the role of age, being adults and young as predictors of retention and attrition in care respectively [11,12]. We found good retention among married and unmarried patients which was similar to a study conducted in Uganda [13]. In contrast to our findings, studies conducted in Ethiopia [14] and Nigeria [15] reported that being unmarried and married were associated with poor retention, respectively.

In our study, we found good retention across all WHO clinical stages, TB/HIV co-infection status, and CD4 count. However, a study conducted in Cameroon [9] and Nigeria [16] reported poor retention among patients in WHO clinical stages I/II/III and clinical stage I and IV respectively. In terms of attrition, a study conducted in Eswatini [17] reported that advanced HIV/AIDS disease (WHO stages III and IV) was significantly associated with attrition. These variable results across WHO disease stages might be explained by different geographic areas and socioeconomic status among studies. In contrast to our findings, another study conducted in Eswatini [17] reported an association of TB/HIV co-infection with attrition whereas no association was found in Indonesia between co-infected patients with attrition [18].

In this study, gender was not associated with virological unsuppression. Contrary to our results, studies conducted in Ghana [19], Morocco [20], and Haiti [21] reported that male patients were less likely to achieve virologic suppression. A possible explanation for virological failure among males may be due to their low health-seeking behavior as compared to females However, a study conducted in Thailand reported female sex as a factor associated with virological failure [22]. This may be due to limited access to care and cultural dynamics, such as patriarchal underpinnings to care-seeking by females in the study environment.

Our analysis revealed that patients´ age was not associated with viral load unsuppression. In contrast to our finding, studies conducted in Ethiopia [23] and Mozambique [24] found that patients´ age < 35 years and younger age were associated with virological unsupression, respectively. Our finding may be due to the strong adherence plan provided to patients at the facility setting, irrespective of their age. Furthermore, our study revealed no association between marital status and virological unsupression. Similar to our finding, a study conducted in Myanmar reported that divorced or separated patients had a lower risk of virological failure [25] whereas a study conducted in Rwanda reported that married/ever married was associated with viral load suppression [26]. No association between marital status and virological unsupression in our cohort may be due to new strategies implemented by the Lesotho Ministry of Health to encourage sexual partners to take ART in one facility as well as referrals of partners to maternal and child health should they need a maternity plan.

Contrary to our finding, studies in Mozambique [24], Rwanda [27], and Uganda [28] found WHO clinical stage as a predictor of ART failure. TB/HIV co-infected patients in our cohort study were not at risk of virological unsupression. Contrary to our finding, studies conducted in South Africa [29] and Haiti [21] reported TB treatment as a risk factor for unsuppressed viral load. The difference in results between these studies may be due to the fact that ART and anti-TB constituted major obstacles for patients. Pill burden and adverse drug-to-drug interaction might have also posed challenges leading to intolerance, poor adherence, and subsequent virological unsupression.

Literature shows that it is difficult to measure adherence in the outpatient setting with absolute precision and accuracy as it may result in recall biases due to its dependence on patients´ self-report. Therefore, researchers used a variety of adherence assessment methods, of which none is considered to be a gold standard [30]. Regarding socio-demographic characteristics, our study has shown that sex, age, and marital status were not associated with ART adherence. This finding is supported by studies conducted in Indonesia [31], Nepal [32], and Brazil [33]. Findings from other studies conducted in Kenya [34] and Ethiopia [35] were in conformity with our study which showed no association of sex, age and adherence, whereas studies conducted in Nigeria [1] and Ethiopia [36] reported good adherence among male patients and patients of 35 - 45 years old, respectively. With regard to marital status. Our finding is in line with studies conducted in Iran [37] and Kenya [38]. Whereas, research from Ethiopia [35] reported that married patients on ART were 3 times more adherent than singles. An explanation for our findings may be due to intensive adherence counseling provided to all adult patients, regardless of their marital status.

In terms of clinical characteristics, this study indicated no association between WHO clinical stage, TB/HIV co-infection, CD4 count, and adherence. Our finding is consistent with a study conducted in Ethiopia [35]. In contrast, studies conducted in south western Ethiopia [39] and in Tanzania [40] reported an association between WHO clinical stage II and more advanced WHO clinical stage with adherence, respectively. Similar to our study, a study conducted in South Africa [41] found no association between TB/HIV co-infection and adherence. However, another study conducted in South Africa [42] reported an association between TB/HIV co-infection and adherence. In regards to CD4 count, our finding is consistent with a study conducted in Iran [37] and Ethiopia [35]. However, studies in Ethiopia [39,43] and in Nigeria [44] reported that CD4 count ≥ 500 mm^3^ were factors significantly associated with good ART adherence and low CD4 count were however associated with poor adherence respectively. Good adherence results in our study may be due to the free ART service and regular counselling strategies implemented by the Lesotho Ministry of Health.

**Limitations:** a major limitation of the study was that we could not follow these patients until 2020 as data was fragmented due to the transition from a paper-based to a digital system (patient-level electronic system) hence we dropped data collection in 2018. Secondly, the nature of this study did not allow us to capture all other factors that may be associated with adherence such as psycho-social, regimen-related issues, socioeconomic status, comorbidities, and adverse events.

**Recommendations:** based on the above findings, we recommend proper data quality management for adequate monitoring of the case cascade; intensification of adherence counselling between 6 months and 1 year; strengthening patient support; and tracing to reduce the number of patients lost to follow-up, as well as to strength HIV testing strategies for early diagnosis of HIV patients.

## Conclusion

We found that retention, viral load suppression and adherence, were generally good among this cohort at 48 months at Sentakana. CD4 at initiation is a predictor of viral load suppression at 48 months.

### What is known about this topic

- *Antiretroviral therapy improves immunity by suppressing HIV viral load;*
- *Monitoring of people living with HIV is mainly based on the assessment of their treatment outcomes;*
- *In Lesotho, HIV prevalence is higher among women than men; it is more than five times higher among young women aged 20-24 years than among their male counterparts. Viral load suppression is sub-optimal*.

### What this study adds

- *Retention, viral load suppression, and adherence were generally good in this cohort at 48 months;*
- *Our study showed no significant association between socio-demographic characteristics and treatment outcome or adherence, except for CD4 at initiation which was a predictor of viral load suppression at 48 months;*
- *Strengthening patient support and tracing to reduce the number of patients lost to follow-up is crucial for HIV programmes; data quality management is important for adequate monitoring of the care cascade at Senkatana HIV Referral Clinic*.
